# Optimizing Young Jackfruit (*Artocarpus heterophyllus* Lam.) Processing for Plant-Based Meatballs: Impact of Thermal Treatments on Quality Parameters and Organoleptic Properties

**DOI:** 10.1155/ijfo/2106508

**Published:** 2025-01-17

**Authors:** Wisutthana Samutsri, Sujittra Thimthuad

**Affiliations:** Food Science and Technology Programme, Faculty of Science and Technology, Phranakhon Rajabhat University, Bangkok, Thailand

**Keywords:** heat treatment, meatball formulation, physicochemical properties, plant-based meat, sensory evaluation, young jackfruit

## Abstract

As global demand for plant-based foods increases due to their nutritional and environmental benefits, young jackfruit (*Artocarpus heterophyllus*) is emerging as a promising meat alternative. This study evaluates the effects of heat treatments—specifically blanching for 5 min and boiling for 15, 30, and 45 min—on the quality and sensory attributes of jackfruit-based meatballs. The results indicate consistent color values (*L*^∗^, *a*^∗^, and *b*^∗^) across the samples, with *L*^∗^ values ranging from 53.68 to 54.92 and *a*^∗^ values from 3.02 to 3.38. The browning index increased with longer boiling times, while the water holding capacity improved from 2.22 to 4.35 as the cooking time extended. Blanching increased the hardness (536.93 g) and springiness (8.30%) of the meatballs. However, these properties decreased with longer boiling times, reaching 317.44 g and 7.68%, respectively, after 45 min. Sensory analysis revealed a strong preference for meatballs made from young jackfruit boiled for 45 min, with the highest score in appearance, flavor, and overall acceptability. These findings suggest that boiling young jackfruit for 45 min optimizes its texture and sensory qualities, highlighting its potential as a sustainable, nutritious, and appealing ingredient for plant-based meat substitutes.

## 1. Introduction

The global shift towards plant-based foods is intensifying due to their nutritional benefits, environmental sustainability, and consumer interest in alternative protein sources. As consumers become more health-conscious and environmentally aware, the demand for plant-based products has surged. Alternative meat products, valued at $4.6 billion in 2018, are projected to reach $85 billion by 2030 [1]. Furthermore, Sha and Xiong [2] estimate a market worth of $30.9 billion by 2026, underscoring rapid growth fueled by those following vegetarian or vegan lifestyles. Plant-based diets offer a viable route to meet nutritional needs while addressing ethical and environmental concerns surrounding traditional animal agriculture.

Young jackfruit (JF) (*Artocarpus heterophyllus*) has gained considerable attention as a versatile meat alternative due to its unique fibrous texture and nutritional profile. This tropical fruit, originating in Western India and now cultivated in various regions, including Asia, Africa, and South America, is a sustainable option that requires minimal maintenance and thrives in diverse environments [3, 4]. Young JF is known for its meat-like texture, making it a popular choice for plant-based diets. It is high in fiber, low in calories, and rich in various vitamins and minerals [5]. The potential of JF as a meat substitute is further supported by its use in traditional dishes and its increasing popularity in modern culinary applications, such as pulled “pork” sandwiches and tacos [6].

Innovative processing methods have been explored to enhance the physicochemical properties of young JF-based meat analogues. For instance, recent research investigated the effects of drying methods, including tray drying and vacuum freeze drying, on chicken meat analogues made from young JF. The findings demonstrated that vacuum freeze drying at lower temperatures preserved superior sensory qualities [7]. The study by Mohammad and Nur [8] found that substituting fat with JF or breadfruit in low-fat chicken patties significantly enhanced water holding capacity (WHC), moisture, and protein levels while reducing fat content. Sensory evaluations indicated consumer preferences for the reformulated chicken patties. Overall, JF proves to be an effective and appealing fat substitute for healthier, low-fat patties. Furthermore, the fibrous structure of young JF contributes to its ability to mimic the texture of animal meat, making it an attractive ingredient for creating plant-based products such as meatballs [9, 10]. Plant-based products can deliver key health benefits, including high dietary fiber and essential vitamins, which can enhance physical performance and overall well-being. Meat alternatives with about 30% protein and low-fat content can effectively replace traditional meats [11]. Research indicates that plant-based meat alternatives can reduce metabolic risks linked to obesity and cardiovascular disease [12, 13] and exhibit anticancer, anti-inflammatory, and immune-enhancing properties [14]. Given these advantages, plant-based foods present a valuable option for consumers aiming to improve their health and physical performance.

Beyond health, traditional livestock farming poses substantial environmental challenges, including greenhouse gas emissions and water pollution. Reducing meat consumption significantly lowers resource use and greenhouse gas output [15]. Reports indicate that components of plant-based meats generate far lower greenhouse emissions than conventional meats, although nutritional content and processing methods may vary [16]. The exploration of alternative raw materials continues, with research focusing on plant-based options that offer meat-like textures and robust nutritional profiles [5, 17–19].

Given the considerable volume of young JF left as waste—up to 120 fruits per mature tree, with only about 15 selected for consumption [4]—this study seeks to utilize young JF by-products to develop plant-based meatballs. Furthermore, the research will investigate how different heat treatments affect the physicochemical and sensory properties of these meatballs, optimizing JF processing for plant-based applications.

## 2. Materials and Methods

### 2.1. Materials

Three young Thai commercial “Thong Prasert” JFs (*Artocarpus heterophyllus* Lam.), approximately 7–11 weeks postflowering, with an average circumference of 45–55 cm, were sourced from a local market in Lopburi Province, Thailand, to serve as independent samples, with one JF used per experimental run. The JFs were categorized by maturity stages, with stage two, corresponding to 9 weeks postflowering, selected as per Konsue, Bunyameen, and Donlao [18]. Soy protein isolate and wheat gluten were obtained from Siam Modified Starch Co., Ltd. (Thailand), and citric acid was sourced from Krungthepchemi Co., Ltd. (Thailand). Additional ingredients approved by the Thai FDA, including sugar, salt, vegetarian seasoning powder, garlic powder, ground pepper, and soybean oil, were sourced from a local market in Bangkok, Thailand.

### 2.2. JF Preparation

**Figure 1:**
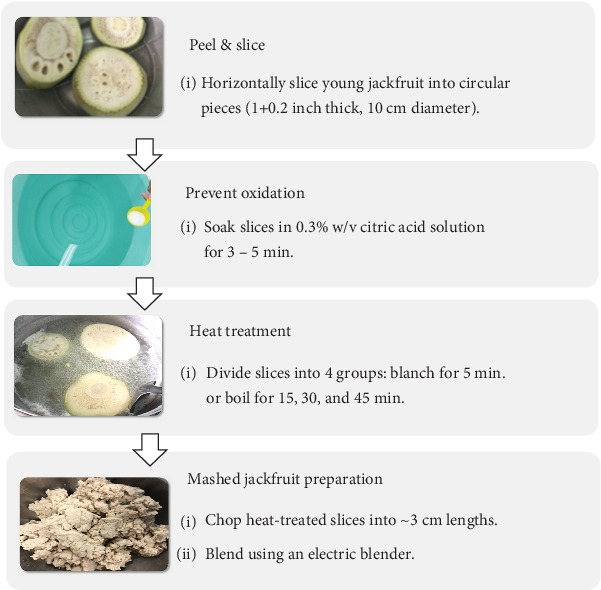
The preparation process of young jackfruit samples. Note: The flowchart was adapted with minor modifications from Samutsri, Oumaree, and Thimthuad [20].

To prepare the young JF, the fruit was first thoroughly cleaned and sliced horizontally with 2 ± 0.5 cm thickness and 10 ± 2 cm diameter (Table 1). Each slice was soaked in a 0.3% w/v citric acid solution for 5 min to prevent oxidation. Heat processing was then applied to the sliced JF, with treatments including blanching for 5 min, followed by boiling for varied durations of 15, 30, and 45 min. After each heat treatment, the JF was immediately immersed in cold water (4°C ± 2°C) to stop further cooking. The cooled samples were then manually squeezed to remove excess water, cut into smaller pieces (approximately 3 ± 3 cm), and blended by using an electric blender (Philips Model HR2051/00/A, 450 W, 1.25 L capacity) at a speed level of 1 for 5 min to achieve a uniform consistency. Three uniformly treated JF samples were individually stored in sealed plastic containers at 4°C ± 2°C. A detailed flowchart for JF preparation is presented in Figure 1.

### 2.3. Plant-Based Meatball Production

**Figure 2:**
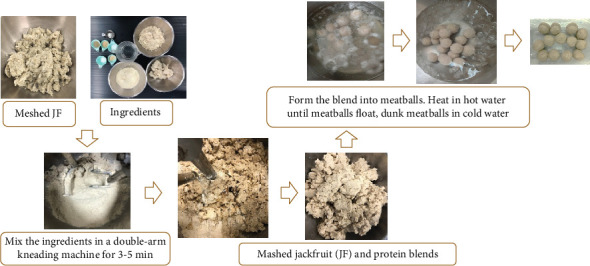
The production of plant-based young jackfruit (JF) meatballs.

The objective of this method was to determine the most suitable heating process for JF in the production of plant-based meatballs. Uniform JF mash from the initial processing stage was utilized to produce the meatballs, following the methodology outlined in Figure 2. Initially, 27 g of uniform JF mash from three different heating treatments (as described in Section 2.2) were equally combined with seasoning and stabilizing ingredients, including sugar, salt, vegetarian seasoning powder, garlic powder, ground pepper, and soybean oil, in the amounts specified in Table 2, according to the method described by Samutsri, Oumaree, and Thimthuad [20]. The mixture was kneaded in a double-arm kneading machine (TONG HOR machine Lex Product 0.85 kW, 0.5–6.0 kg/batch of capacity) for approximately 10 min to ensure thorough homogenization, after which it was manually formed into globular-shaped meatballs with an approximate diameter of 2.5 ± 0.2 cm. These meatballs were then immersed in hot water at 60°C–65°C for 20 min for cooking. Once the meatballs floated to the surface, they were immediately removed from the warm water and transferred to cold water at 4°C ± 2°C for at least 10 s to halt the cooking process. Finally, the meatballs were stored in a plastic container in the refrigerator as the final product until further analysis.

### 2.4. Characterizations of Plant-Based Meatballs

#### 2.4.1. Color Measurement

The color analysis of meshed JF paste and JF meatballs, subjected to three different heat treatments as detailed in Sections 2.2 and 2.3, was performed. For the mashed JF, three samples weighing 3 g each were prepared, resulting in a total of nine samples. The JF meatballs included one piece per treatment with three replicates each, also totalling nine samples. All 18 samples were placed in Petri dishes and sealed with single-use transparent plastic film to facilitate color readings with the colorimeter probe. Color evaluations were conducted using a colorimeter (Minolta, model CR-10, Japan), with readings taken from the surface of each sample. Assessments were performed in triplicate according to the method outlined by Maisont et al. [21]. The color parameters were reported in Commission Internationale de l'Éclairage (CIE) chromaticity coordinates: lightness (*L*^∗^), redness (*a*^∗^), and yellowness (*b*^∗^). Lightness (*L*^∗^) ranged from 0 (*black*) to 100 (*white*). Redness (*a*^∗^) exhibited positive values for red undertones and negative values for green undertones, while yellowness (*b*^∗^) displayed positive values for yellow undertones and negative values for blue undertones. The colorimeter was calibrated using a standard white porcelain plate.

Browning indexes (BIs) were calculated using the *L*^∗^, *a*^∗^, and *b*^∗^ values to assess color changes relative to the control meatball sample. The BI was computed using the following formula [22]:

 $$\begin{array}{c} \text{Browning index }\left(\text{BI}\right)=100\frac{\left(x-0.31\right)}{0.172} \end{array}$$

where *x* = (*a*^∗^ + 1.75 *L*^∗^)/(5.645 *L*^∗^ + *a*^∗^ − 3.012*b*^∗^) *x* = (5.645 × *L*^∗^ − 3.012 × *b*^∗^).

#### 2.4.2. WHC Measurements

WHC was analyzed according to the method of Dobson, Laredo, and Marangoni[23], with a modification to accommodate 2 g of sample mixed with 20 mL of distilled water in a 50-mL centrifuge tube. The slurry was allowed to stand for 10 min and then centrifuged at 6000 × g for 20 min. After centrifugation, the supernatant was drained, and the wet sample residue was weighed. The result was expressed as the amount of water the sample could hold. All samples were analyzed in triplicate. The WHC was calculated using the following equation:

 $$\begin{array}{c} \text{Water holding capacity}=\left(\frac{\text{weight of hydrated sample}-\text{weight of sample}}{\left(\text{weight of sample}\right)}\right) \end{array}$$

#### 2.4.3. Textural Measurements

Texture profile analysis (TPA) was performed on plant-based meatball samples subjected to various young JF processing treatments using a CTX Texture Analyzer (CTX50K, Brookfield, United States). Samples were positioned on a standard fixture on the analyzer table. Test conditions were as follows: Each sample underwent double compression with a TA36 probe (36 mm diameter flat cylinder) and a trigger force of 10 × g. The pretest, test, and posttest speeds were set to 10, 1, and 10 mm/s, respectively, with a compression distance of 6 mm at ambient temperature. At least 10 replicates were measured per treatment. TPA curves were generated for each sample, and textural parameters including hardness, springiness, and chewiness were analyzed and defined according to established criteria [23, 24].

$$\begin{array}{cc} \; & \text{Hardness }\left(\text{g}\right)=\text{maximum peak force of the first compression}\\ \text{Springiness }\left(\%\right)=\text{ratio of maximum peak force of the first compression and maximum peak force of the second compression}\times 100\\ \text{Chewiness}=\text{hardness}\times \text{cohesiveness}\times \text{springiness} \end{array}$$

### 2.5. Sensory Evaluation

The sensory evaluation for optimizing the plant-based meatball formulation involved assessing samples with different heat-treated young JF variations. Fifteen semitrained panellists aged 18–22, participated in individual booths within the sensory laboratory of the Food Science and Technology program at Phranakhon Rajabhat University. Panellists were instructed on using a seven-point hedonic scale to rate each attribute: appearance, color, taste, flavor, texture, and overall liking, where 1 represented “*dislike extremely*” and 7 represented “*like extremely*.” Data were analyzed using analysis of variance (ANOVA) with blocked randomization in Statistical Package for the Social Sciences (SPSS) Statistics (Version 18.0). The final plant-based meatball formulation was selected based on these sensory results.

### 2.6. Statistical Analysis

All experiments were conducted in triplicate. Mean values and standard deviations were calculated for each set of results. A one-way ANOVA was performed using SPSS Statistics (Version 18.0) to assess differences among treatments. Significant differences between mean values (*p* ≤ 0.05) were determined using Duncan's multiple range test.

## 3. Results and Discussions

### 3.1. Characteristics of Young JF

Young JF harvested in this study was selected during the second growth stage, from Weeks 7 to 11, which corresponds to the optimal period for developing desirable physical and textural qualities for culinary applications (Table 1). At this stage, each fruit exhibited an average weight of 2500–3000 g, with a circumference of 45–55 cm and a length of 60–70 cm, as shown in Table 1. These size parameters are significant for both yield estimation and processing requirements.

Each horizontal slice of young JF, approximately 1 ± 0.2 inches thick, contained an average of 8 to 10 seeds, which contributes to the textural profile important in product formulation. The usable flesh yield per fruit ranged from 1100 to 1284 g, representing 42.8%–44.0% of the total fruit weight. This yield percentage is particularly relevant for food processing applications, where maximizing edible portion is critical for commercial viability.

These findings suggest that young JF, harvested within this specific growth window, offers a consistent and substantial yield of usable flesh, supporting its potential as a plant-based ingredient. The high flesh yield, coupled with uniform physical characteristics, underscores the fruit's suitability for processed food products, aligning with consumer demand for plant-based alternatives.

### 3.2. Formulation of Young JF-Based Plant-Based Meatballs

**Figure 3:**
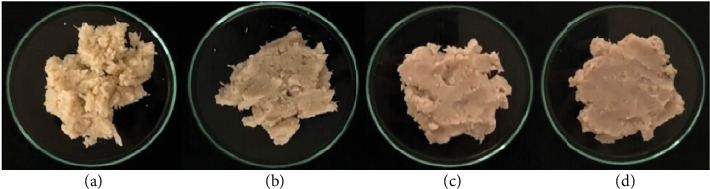
Photographic representation of the appearance, color, and texture of 3 g of mashed young jackfruit on a Petri dish subjected to various heat treatments: blanched for (a) 5 min and boiled for (b) 15 min, (c) 30 min, and (d) 45 min, before color measurement analysis.

The formulation and processing of plant-based meatballs were optimized based on the sensory characteristics, as summarized in Table 2. Young JF was subjected to scalding or boiling at various times before being ground into a paste to produce the meatballs (Figure 3).

In developing plant-based meatballs, various measures were implemented to ensure product consistency. Young JF was selected for its ability to mimic the texture of pork [25]. Typically, soy and its derivatives dominate meat substitutes; however, the inclusion of a mixture of wheat flour, corn flour, and powdered rice flakes provides a necessary fiber boost to maintain gastrointestinal health [26, 27]. Wheat gluten plays a crucial role in producing meat analogues, acting as both a binding agent and a structural component by forming fibrous textures under mechanical deformation [11, 27].

### 3.3. Color and BI of Plant-Based Meatballs Made From Young JF

**Figure 4:**
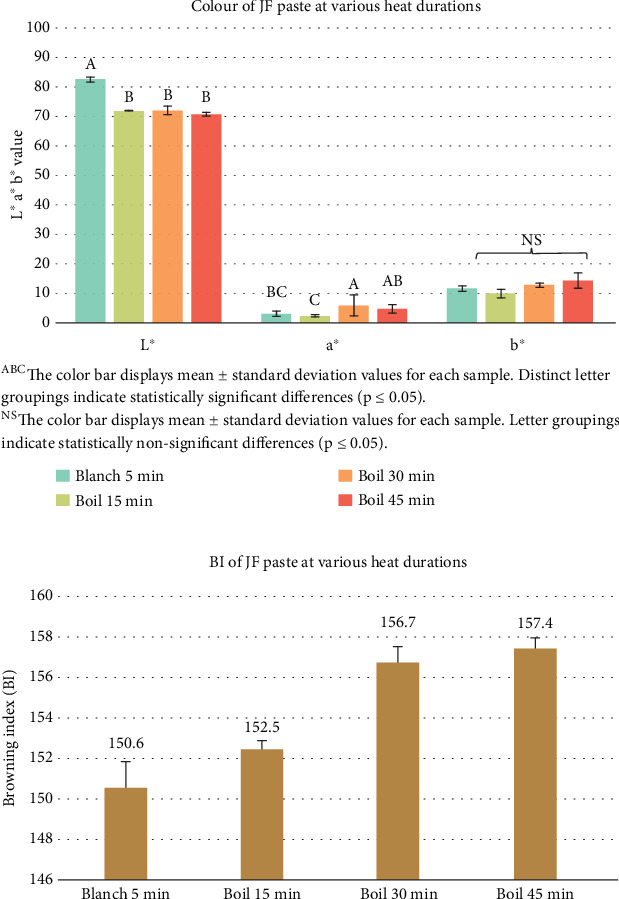
(a) Color values and (b) calculated browning index of mashed jackfruit (JF) subjected to varying durations of heat treatment.

Various heat treatments, including blanching for 5 min and boiling for 15, 30, and 45 min, were applied to the JF. Significant differences (*p* ≤ 0.05) in color values of JF pastes were observed across the heat treatments (Figure 4(a)). The *L*^∗^ values ranged from 70.80 to 82.53, *a*^∗^ values ranged from 2.30 to 5.73, and *b*^∗^ values ranged from 9.93 to 14.20. The BI was lowest for blanched samples and increased with boiling time (Figure 4(b)), indicating the extent of the Maillard reaction and caramelization processes at higher temperatures.

**Figure 5:**
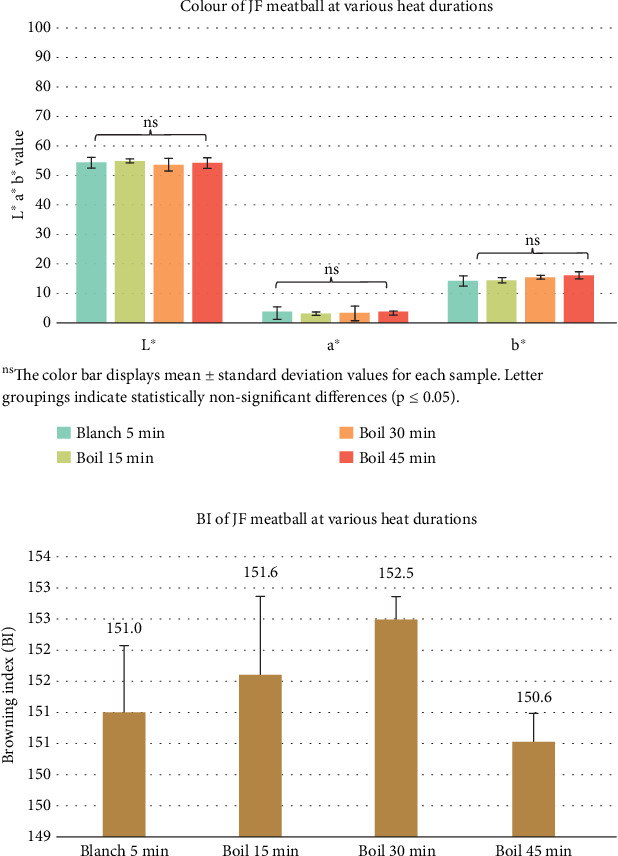
(a) Color values and (b) calculated browning index of plant-based meatballs made from jackfruit (JF) subjected to varying durations of heat treatment.

The color values of the meatballs showed no significant differences (*p* > 0.05) across heat treatments (Figure 5(a)), with average *L*^∗^ values ranging from 53.68 to 54.92, *a*^∗^ values ranging from 3.02 to 3.38, and *b*^∗^ values ranging from 14.18 to 16.04. The BI also demonstrated no significant changes (*p* > 0.05) (Figure 5(b)). Although the heat treatments significantly impacted the mashed JF color (Figure 4), the final meatballs exhibited consistent color values. This consistency suggests that the meatball matrix's structure and composition mitigated the visual effects of browning, allowing the inherent qualities of JF to prevail in the final product.

### 3.4. WHC of Young JF-Based Plant-Based Meatballs

The WHC of the meatballs was significantly influenced by the heat treatments (*p* ≤ 0.05) (Table 3). Extended processing times led to higher WHC, attributed to the solubilization of pectin in young JF, which forms a gel-like structure capable of entrapping water [28]. This moisture retention is crucial for enhancing product juiciness and overall sensory experience, aligning with consumer expectations for desirable meat substitutes.

### 3.5. Textural Characteristics of Young JF-Based Plant-Based Meatballs

TPA is a technique used to simulate the biting actions of the mouth through a two-compression process [24]. This method involves using a device that measures the chewing mechanism by applying a pressure probe twice. This texture profile illustrates the relationship between force and time. Significant differences in texture profile characteristics are crucial for consumer acceptance. Achieving a balanced relationship between hardness, springiness, and chewiness is crucial for creating a texture that mimics traditional meat. This is particularly important for plant-based and imitation meat products that are aimed at replicating the eating experience of real meat, aligning with consumer expectations.

Hardness is the force required to break or separate the food. This measurement is obtained by pressing the product between the molars or between the tongue and the roof of the mouth. The maximum pressure recorded during this process determines the hardness value of the sample [23]. Hardness significantly decreased with extended heating, from 536.93 g for blanched samples to 317.44 g for those boiled for 45 min (*p* ≤ 0.05) (Table 4). The observed effect is due to the heat extraction of the pectin structure in young JF, which serves as the primary structural component of meatballs. According to Saxena, Bawa, and Raju [29], JF is an excellent source of pectin. Consequently, the gel-like structure formed by pectin in the JF pulp reaches its peak when heated for an extended period, resulting in the lowest hardness value of the meatballs [28]. Moreover, this reduction correlates with increased moisture content, enhancing the tenderness of samples [30]. Silva et al. [31] reported that as the moisture content in the extrusion feed increased, the hardness of soy protein–based high-moisture meat analogues decreased. This finding supports the observation that higher levels of heating result in reduced hardness, which correlates with the increased WHC noted at elevated heating levels (as illustrated in Table 3).

Springiness is defined as the rate at which a material returns to its shape after being pressed twice, measured by the distance between them. The results exhibited significant differences, with blanched meatballs showing the highest springiness values (8.30%), gradually declining with increased boiling time (*p* ≤ 0.05) (Table 4). This trend suggests that while short heat treatments can enhance the springiness of the meatball structure, prolonged boiling may lead to the extraction of structural components like pectin, which diminishes overall flexibility. The mechanical properties of plant fiber/soy protein adhesive composites mainly depended on the morphology of the plant fiber, the content of the main components of fiber, and the interfacial addition between plant fiber and protein adhesives [32]. As the heating level increased, the springiness of the young JF decreased due to its high WHC. This resulted in a loose and soft product that provided minimal resistance during the compression test. This observation aligns with the findings of Taikerd and Leelawat [33].

Chewiness is defined as the energy required to masticate food until it can be swallowed. The chewability of meatballs made with young JF subjected to different heat treatment durations was analyzed. The average values of chewiness showed no significant differences across treatments (*p* > 0.05) (Table 4), indicating that the formulations maintained consistent chewability despite variations in hardness and springiness.

### 3.6. Sensory Evaluation of Young JF-Based Plant-Based Meatballs

**Figure 6:**
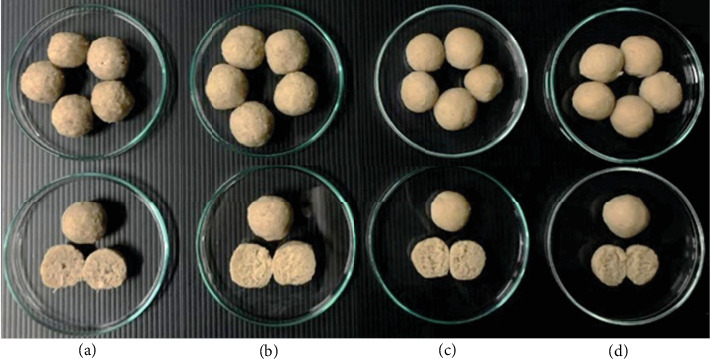
The appearance of plant-based meatballs made with young jackfruit, each with a globular shape approximately 2.5 cm in diameter. Images include a cross-section to display the internal texture of samples subjected to different heat treatments: blanched for (a) 5 min and boiled for (b) 15, (c) 30, and (d) 45 min.

The sensory characteristics of the meatballs were evaluated based on the appearance of plant-based meatballs made from young JF. These meatballs underwent different heat treatments: blanched for 5 min and boiled for 15, 30, and 45 min, as illustrated in Figure 6.

**Figure 7:**
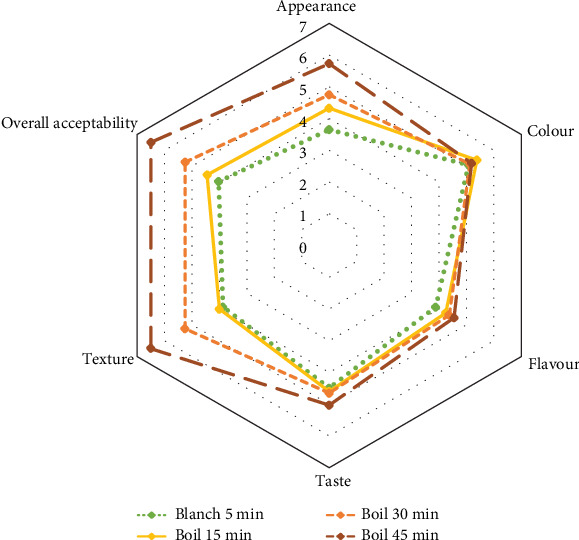
Radar chart of sensory evaluation scores for plant-based meatballs from young jackfruit under varying heat treatment durations.

**Figure 8:**
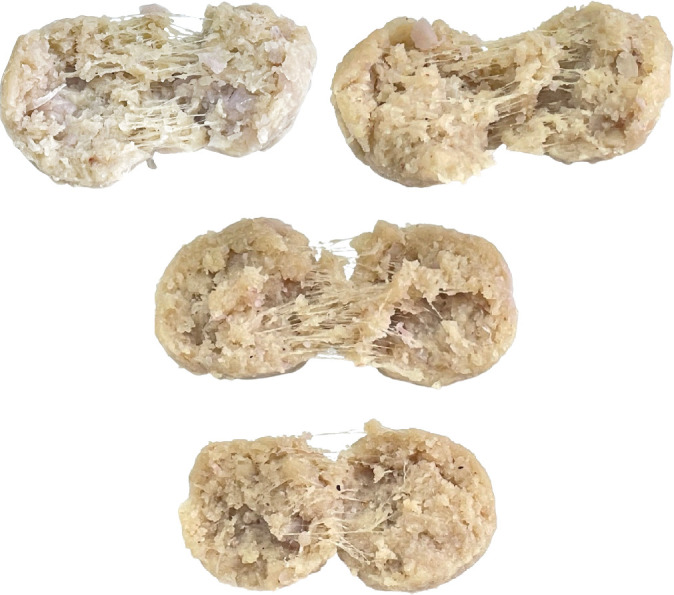
Close-up photographs illustrating the internal characteristics, including texture, color, and fiber structure, of young jackfruit-based plant-based meatballs subjected to different heat treatments: blanched for (a) 5 min and (b) boiled for 15, (c) 30, (d) and 45 min.

Based on the sensory evaluation results for each characteristic (Figure 7), meatballs made with young JF boiled for 45 min received the highest liking scores for appearance, color, flavor, taste, texture, and overall satisfaction. The preference for these meatballs can be attributed to their nonfibrous texture, in contrast to those boiled for shorter durations, such as 30 min (Figure 8).

This study evaluated the quality of meatballs made from young JF subjected to different heat treatments. Blanching for 5 min or boiling for 15, 30, and 45 min impacted their physical properties, including color (*L*^∗^, *a*^∗^, and *b*^∗^ values), texture characteristics (hardness, springiness, chewiness), and sensory quality. Color is a primary sensory attribute that influences the initial appeal of food products. In this study, while heat treatments significantly impacted the color values of JF pastes, the final meatball color remained consistent across treatments. This stability ensures a uniform product appearance, essential for consumer expectations of meat substitutes. Notably, meatballs made from young JF boiled for 15 min exhibited desirable color and flexibility. In contrast, the sensory evaluation revealed that panellists rated the meatballs boiled for 45 min as the best across all attributes. Longer boiling time enhances juiciness and tenderness by increasing WHC and reducing hardness; however, excessive boiling led to a decline in springiness, indicating a trade-off between moisture retention and structural flexibility. Reduced hardness and springiness at higher WHC levels, which is a key sensory attribute, align with consumer preferences for moist and tender products. The study indicates that the optimizing heat treatment achieves a balance between improved juiciness and tenderness while maintaining acceptable texture and structural integrity, which meets sensory expectations for plant-based meat alternatives. Thus, meatballs made from young JF that were heat-treated by boiling for 45 min were chosen as the optimal product.

## 4. Conclusions

This study evaluated the impact of varying heat treatment durations on the physicochemical and sensory attributes of young JF in plant-based meatball formulations. Heat treatments included blanching for 5 min and boiling for 15, 30, and 45 min to assess their effects on product quality. Results indicated that heating duration significantly influenced the color and BI of JF paste, though these changes did not persist in the final meatball product. Extended heating times improved WHC due to pectin extraction, forming gel-like structures that resulted in softer textures. Sensory evaluation showed that meatballs boiled for 45 min were preferred across all attributes—appearance, color, flavor, taste, texture, and overall acceptability—primarily due to reduced fibrousness. These findings offer valuable insights for optimizing processing conditions for JF-based products in the plant-based meat industry.

## Figures and Tables

**Table 1:** Physical characteristics of fresh young jackfruit.

**Characteristics**	**Measurement**
Growth stage (weeks)	7–11
Weight per fruit (g)	2500–3000
Circumference (cm)	45–55
Length (cm)	60–70
Number of seeds per 2 ± 0.5 cm slice	8–10
Yield (%)	42.8–44.0

**Table 2:** Formulation of young jackfruit-based plant-based meatballs in this study.

**Ingredients**	**Content (g)**
Jackfruit	27.0
Soy protein isolate	13.5
Wheat gluten	12.7
Sugar	0.5
Salt	0.6
Vegetarian seasoning powder	0.3
Garlic powder	0.3
Ground pepper	0.2
Soybean oil	0.6
Water	44.3

*Note:* Adapted from Samutsri, Oumaree, and Thimthuad [20].

**Table 3:** Average water holding capacity of plant-based meatballs made from young jackfruit at various heat treatment durations.

**Heating process**	**Water holding capacity**
Blanch 5 min	2.65 ± 0.25^b^
Boil 15 min	2.22 ± 0.71^b^
Boil 30 min	4.35 ± 1.59^a^
Boil 45 min	3.69 ± 1.01^ab^

*Note:*Mean ± standard deviation values in the same column with different superscript letters are significantly different (*p* ≤ 0.05).

**Table 4:** Textural characteristics of plant-based meatballs made from young jackfruit under different heat treatment durations.

**Heating process**	**Hardness (g)**	**Springiness (%)**	**Chewiness** ^**#**^
Blanch 5 min	536.93 ± 43.32^a^	8.30 ± 0.48^a^	27.13 ± 2.48
Boil 15 min	445.20 ± 33.20^b^	7.84 ± 0.26^b^	28.39 ± 3.50
Boil 30 min	380.43 ± 38.05^c^	7.97 ± 0.31^ab^	25.97 ± 3.21
Boil 45 min	317.44 ± 31.87^d^	7.68 ± 0.26^b^	25.06 ± 3.29

*Note:*Mean ± standard deviation values in the same column with different superscript letters are significantly different (*p* ≤ 0.05).

^#^Mean ± standard deviation in the same column with different superscript letters is nonsignificantly different (*p* ≥ 0.05).

## Data Availability

The original contributions presented in the study are included in the article material. Further inquiries can be directed to the corresponding author.
